# Incidence of and Factors Associated with Spontaneous Correction of Postoperative Shoulder Imbalance in Adolescent Idiopathic Scoliosis Patients: A Retrospective Cohort Study

**DOI:** 10.1007/s43465-024-01293-2

**Published:** 2024-12-09

**Authors:** Pakorn Chawanpaiboon, Surin Thanapipatsiri, Chatupon Chotigavanichaya, Sirichai Wilartratsami, Monchai Ruangchainikom, Ekkapoj Korwutthikulrangsri

**Affiliations:** Department of Orthopaedic Surgery, Faculty of Medicine Siriraj Hospital, Mahidol University, 2 Wanglang Road, Bangkoknoi, 10700 Bangkok Thailand

**Keywords:** Adolescent idiopathic scoliosis, Associated factors, Postoperative shoulder imbalance, Regression analysis, T1 tilt

## Abstract

**Background:**

As far as we know, no study has investigated spontaneous postoperative shoulder imbalance (PSI) correction in adolescent idiopathic scoliosis (AIS) patients. The objective is to assess the incidence of and associated factors for spontaneous PSI correction in AIS patients.

**Methods:**

The study evaluated 144 postoperative AIS patients with PSI aged 10–20 years between 2010 and 2018. An analysis included demographic data and radiographic measurements (Risser grading, Lenke type, upper instrumented vertebra [UIV], and lowest instrumented vertebra [LIV]). Preoperative, postoperative, and follow-up radiographs were evaluated for shoulder parameters: radiologic shoulder height (RSH); T1 tilt angle; clavicle angle (CA); proximal thoracic curve (PTC), main thoracic curve (MTC), and lumbar curve (LC) Cobb measurements; and apical vertebral translation (AVT) of the PTC, MTC, and LC.

**Results:**

Spontaneous PSI correction was observed in 99 (68.75%) patients. The spontaneous correction and nonspontaneous correction groups differed significantly in terms of Lenke-type preoperative LC (23° vs 26°; P = 0.091), postoperative LC (11° vs 8°; P = 0.013), LC at follow-up (13.5° vs 9°; P = 0.028), postoperative AVT of LC (− 0.8° vs − 0.4°; P = 0.033), AVT of LC at follow-up (− 0.7° vs − 0.1°; P = 0.091), PTC at follow-up (16° vs 20°; P = 0.019), and AVT of PTC at follow-up (0° vs -0.3°; P = 0.029). Multivariate analysis identified postoperative T1 tilt and postoperative LC as significantly associated with PSI correction.

**Conclusions:**

The incidence of spontaneous PSI correction is high. Postoperative T1 tilt and postoperative LC are significantly associated with spontaneous PSI correction.

## Introduction

Postoperative shoulder imbalance (PSI) is prevalent in adolescent idiopathic scoliosis (AIS) patients undergoing surgery, causing poor cosmesis and patient dissatisfaction [1]. The incidence of PSI is approximately 25%, with Lenke types 2 and 5 AIS being commonly associated with PSI [2].

Radiologic shoulder height (RSH), clavicle angle (CA), and T1 tilt angle are parameters used to define PSI, with RSH being the most frequently used [3, 4]. However, there is still controversy over the definition of PSI. While some studies define PSI as an RSH greater than 10 mm [5, 6], Akel et al. found that some healthy adolescents also had an RSH over 10 mm [7]. Therefore, an RSH greater than 20 mm was used in several studies to define PSI [8–10].

Many factors have been studied for their association with PSI. They include Cobb angle; apical vertebral translation (AVT) of the proximal thoracic curve (PTC), main thoracic curve (MTC), and lumbar curve (LC); coronal shift; and Risser grading [2, 8, 11].

**Fig. 1:**
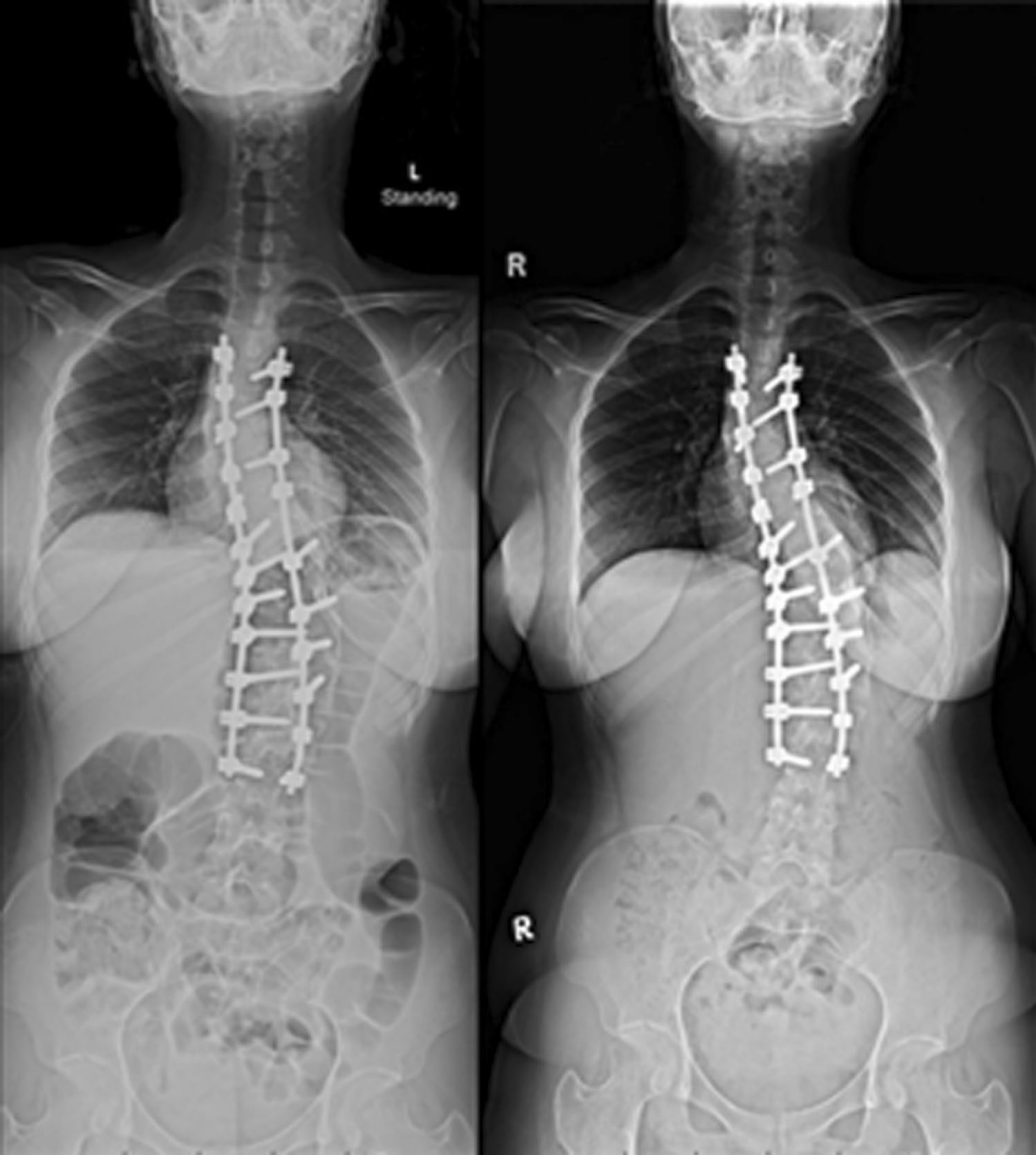
An example of spontaneous correction of PSI. (Left) Immediate postoperative standing whole-spine frontal radiograph of an AIS patient revealed PSI. (Right) Whole-spine frontal radiograph at 1 year follow-up in the same patient revealed spontaneous correction of PSI

**Fig. 2:**
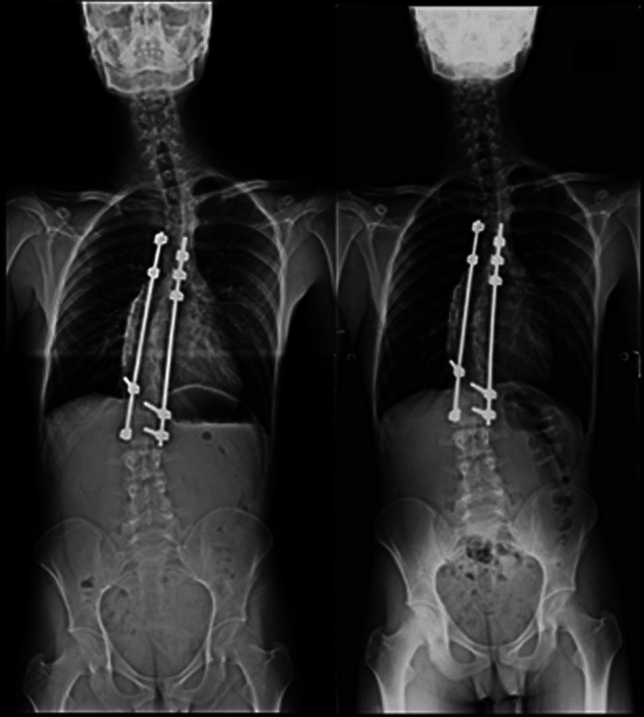
An example of no-correction of PSI. (Left) Immediate postoperative standing whole-spine frontal radiograph of an AIS patient revealed PSI. (Right) Whole-spine frontal radiograph at 1 year after operation in the same patient revealed no correction of PSI

We have observed spontaneous correction of PSI in some patients, with the shortest follow-up time being 1 month postoperatively (Figs. 1, 2). However, no previous studies have investigated the spontaneous correction of PSI. The present study aimed to determine the incidence and associated factors for spontaneous correction of PSI.

## Materials and Methods

### Design and Patient Selection

A total of 144 AIS patients who underwent corrective surgery between January 2011 and December 2018 at our hospital were included in this study. The inclusion criteria were AIS patients aged 10 to 20 years who underwent corrective surgery using a posterior approach. This study was approved by the hospital’s institutional review board of the Faculty of Medicine, Siriraj Hospital (study no. Si 622/2020). The exclusion criteria were as follows:

- Revision surgery
- The use of Harrington distraction instrument or sublaminar wiring
- Absence of postoperative whole-spine radiograph frontal at 1–12 months after surgery
- Evidence of hip pathology (eg, slipped capital femoral epiphysis, hip fracture, Legg–Calvé–Perthes disease, tumor)
- Evidence of spondylolisthesis
- Evidence of limb length discrepancy

### Data Collection and Statistical Analysis

A record was made of demographic data (age and sex), radiographic measurements (Risser grade at surgery, Lenke type, upper instrumented vertebra [UIV], lowest instrumented vertebra [LIV]), and follow-up time. Whole-spine anteroposterior radiographs were taken preoperatively, immediately postoperatively, and at follow-up. The Cobb method was used to evaluate PTC, MTC, and LC. Assessments were also made of the AVT of the PTC, MTC, and LC; coronal shift; UIV tilt; and LIV tilt [12].

RSH, T1 tilt angle, and CA were used to assess PSI. RSH is the difference in soft tissue shadow directly superior to the acromioclavicular joint [13]. CA is the angle between a horizontal line and a line connecting the highest point of the clavicle. T1 tilt is the angle between a horizontal line and a line along the superior endplate of T1 [7]. PSI was defined as an RSH ≥ 20 mm with at least one of the following measures: (1) T1 tilt ≥ 3 degrees; (2) CA ≥ 3 degrees. Spontaneous correction of PSI was noted if the follow-up radiograph no longer met the PSI criteria.

The UIV tilt angle is the angle between the horizontal line and the superior endplate line of the UIV [14]. The LIV tilt angle is the angle between the horizontal line and the inferior endplate line of the LIV [15].

The mean ± standard deviation (SD) was used to report continuous data with normal distribution. The median (interquartile range; Q1-Q3) was used to report continuous data with non-normal distribution. Univariate analysis was used to identify predictors of spontaneous correction of PSI. Multivariate logistic regression was performed on significant factors (*P* < 0.2) to identify independent factors for spontaneous correction of PSI. Receiver-operating characteristic (ROC) analysis was used to evaluate the discriminatory effect of factors associated with the correction of PSI, with AUC used to measure the logistic regression model’s capacity for discrimination. The author performed the measurement twice, with a 1-week interval, to evaluate the degree of intra-rater reliability. The intra-rater and inter-rater reliability were calculated using the intraclass correlation coefficient (ICC). The ICC value was interpreted as followed: 0.00–0.20, poor agreement; 0.21–0.40, fair agreement; 0.41–0.60, moderate agreement; 0.61–0.80, good agreement; and 0.81–1.00, excellent agreement [16]. The ICCs for intra-rater and inter-rater agreement were above 0.9 for all measurements.

## Results

The study analyzed 144 patients, of whom 120 (83.33%) were female. The mean age at surgery was 14.71 ± 1.99 years, and the mean follow-up time was 8.47 ± 3.57 months. Lenke type 1 AIS was the most common (34.72%), while the most frequent Risser grade was grade 4 (45.83%). Spontaneous PSI correction was observed in 99 (68.75%) patients. No significant differences were found in the demographic data, Risser grades, Lenke types, and follow-up times of the spontaneous and nonspontaneous correction groups (*P* > 0.05) (Table 1).

**Table 1:** Demographic characteristics of spontaneous correction and no-correction of PSI

	Correct		*P* value
	Yes (*n* = 99, 68.75%)	No (*n* = 45, 31.25%)	
Age (years)^†^	14.8 ± 2.1	14.5 ± 1.7	0.479
Gender (%) Female Male	83 (83.8) 16 (16.2)	37 (82.2) 8 (17.8)	0.813
Risser grading (%) 0 1 2 3 4 5	3 (3) 2 (2) 10 (10.1) 20 (20.2) 44 (44.4) 20 (20.2)	1 (2.2) 0 (0) 2 (4.4) 8 (17.8) 22 (48.9) 12 (26.7)	0.703
Lenke type (%) 1 2 3 4 5 6	31 (31.3) 18 (18.2) 12 (12.1) 9 (9.1) 25 (25.3) 4 (4.0)	19 (42.2) 11 (24.4) 5 (11.1) 3 (6.7) 6 (13.3) 1 (2.2)	0.515
Follow-up time (months)^‡^	9 (6–12)	10 (6–12)	0.493

^†^Mean ± SD, ^‡^Median(Q1-Q3)

Statistically significant differences existed between the spontaneous correction and the no-correction groups concerning Lenke-type preoperative LC (23° vs 26°; *P* = 0.091), postoperative LC (11° vs 8°; *P* = 0.013), LC at follow-up (13.5° vs 9°; *P* = 0.028), postoperative AVT of LC (− 0.8° vs − 0.4°; *P* = 0.033), AVT of LC at follow-up (− 0.7° vs − 0.1°; *P* = 0.091), PTC at follow-up (16° vs 20°; *P* = 0.019), and AVT of PTC at follow-up (0° vs − 0.3°; *P* = 0.029). However, there were no significant differences between the spontaneous and nonspontaneous correction groups regarding all other preoperative, postoperative, and last follow-up radiographic measurements (Table 2).

**Table 2:** Radiographic parameters of spontaneous correction and no-correction of PSI

	Spontaneous correction of PSI		*P* value
	Yes (*n* = 99, 68.75%)	No (*n* = 45, 31.25%)	
Preoperative PTC Cobb angle (°)^‡^	23.0 (8.5–31.3)	26.0 (19.0–34.5)	0.091
Preoperative MTC Cobb angle (°)^†^	51.8 ± 22.8	53.6 ± 17.6	0.633
Preoperative LC Cobb angle (°)^‡^	40.0 (31.0–52.0)	32.0 (27.0–45.5)	0.014
Preoperative coronal shift (cm)^†^	0.2 ± 1.9	0.2 ± 1.7	0.961
RSH (cm)			
Preoperative^‡^	1.1 (0.6–2.0)	1.2 (0.7–1.8)	0.997
Postoperative^‡^	2.0 (1.5–2.5)	2.0 (1.6–2.8)	0.515
Last follow-up^‡^	0.8 (0.4–1.1)	1.9 (1.6–2.2)	< 0.001
T1 tilt (°)			
Preoperative^‡^	0 (− 4.0–4.0)	0 (− 2.0–3.0)	0.916
Postoperative^‡^	4.0 (− 2.0–9.0)	6.0 (4.0–8.5)	0.031
Last follow-up^‡^	4.0 (0–7.0)	7.0 (3.0–9.0)	0.011
CA (°)			
Preoperative^‡^	− 2.0 (− 3.0–1.0)	− 2.0 (− 3.0–0)	0.871
Postoperative^‡^	3.0 (− 3.0–5.0)	4.0 (3.0–5.5)	0.146
Last follow-up^‡^	1.0 (− 1.0–2.0)	3.0 (3.0–4.0)	< 0.001
UIV tilt (°)			
Postoperative^‡^	0 (− 7.0–5.0)	− 3.0 (− 7.5–3.0)	0.587
Last follow-up^†^	− 1.4 ± 10.7	− 2.3 ± 7.8	0.554
LIV tilt (°)			
Postoperative^†^	0.1 ± 7.2	0.6 ± 5.5	0.667
Last follow-up^†^	− 0.5 ± 8.0	− 0.2 ± 5.3	0.764
PTC (°)			
Postoperative^‡^	17.0 (9.0–25.0)	22.0 (14.0–27.5)	0.086
Last follow-up^‡^	16.0 (8.0–23.0)	20.0 (14.0–29.5)	0.019
PTC AVT (cm)			
Postoperative^‡^	− 0.1 (− 0.5–0.2)	− 0.4 (− 0.8–0.1)	0.059
Last follow-up^‡^	0 (− 0.4–0.2)	− 0.3 (− 0.6–0.1)	0.029
MTC (°)			
Postoperative^‡^	18.0 (11.0–28.0)	15.0 (10.0–21.0)	0.221
Last follow-up^‡^	19.0 (11.0–30.0)	16.0 (10.0–20.5)	0.214
MTC AVT (cm)			
Postoperative^†^	1.0 ± 2.2	0.7 ± 1.5	0.414
Last follow-up^‡^	0.6 (0–1.7)	0.6 (-0.2–2.0)	0.569
LC (°)			
Postoperative^‡^	11.0 (5.0–20.0)	8.0 (3.0–14.5)	0.013
Last follow-up^‡^	13.5 (5.0–22.0)	9.0 (3.0–15.0)	0.028
LC AVT (cm)			
Postoperative^†^	− 0.8 ± 1.6	− 0.4 ± 1.0	0.033
Last follow-up^‡^	− 0.7 (− 1.6–0.1)	− 0.1 (− 0.95–0.3)	0.025
Coronal shift (cm)			
Postoperative^†^	-0.6 ± 2.3	− 0.8 ± 1.4	0.657
Last follow-up^‡^	− 0.8 (− 1.6–0)	− 0.5 (− 1.6–0.1)	0.516

*PTC* proximal thoracic curve, *MTC* main thoracic curve, *LC* lumbar curve, *RSH* radiologic shoulder height, *CA* clavicle angle, *UIV* upper instrumented vertebra, *LIV* lowest instrumented vertebra, *AVT* apical vertebral translation, ^†^Mean ± SD, ^‡^Median(Q1-Q3)

In the univariate analysis, factors significantly associated with spontaneous correction of PSI were Lenke type 5 (odds ratio [OR]: 2.55; 95% confidence interval [CI] 0.89–7.36; *P* = 0.082), postoperative PTC (OR: 0.97; 95% CI 0.94–1.01; *P* = 0.124), postoperative MC (OR: 1.02; 95% CI 0.99–1.05; *P* = 0.134), postoperative LC (OR: 1.05; 95% CI 1.01–1.10; *P* = 0.014), postoperative AVT of LC (OR: 0.78; 95% CI 0.60–1.29; *P* = 0.070), postoperative CA (OR: 0.52; 95% CI 0.23–1.18; *P* = 0.118), and postoperative T1 tilt (OR: 0.29; 95% CI 0.11–0.76; *P* = 0.012).

In the multivariate analysis, the adjusted ORs of spontaneous correction of PSI were statistically significant for postoperative LC (OR: 1.04; 95% CI 1.00–1.09; *P* = 0.049) and postoperative T1 tilt (OR: 0.36; 95% CI 0.14–0.96; *P* = 0.041) (Table 3).

**Table 3:** Multiple logistic regression analysis of spontaneous correction of PSI

	Odds ratio	95% confidence interval	*P* value
Postoperative T1 tilt	0.3602	0.1353–0.9590	0.041
Postoperative LC	1.0434	1.0003–1.0884	0.049
Constant	2.9854	1.0462–8.5193	0.041

*LC* lumbar curve, Constant estimates baseline odds

**Fig. 3:**
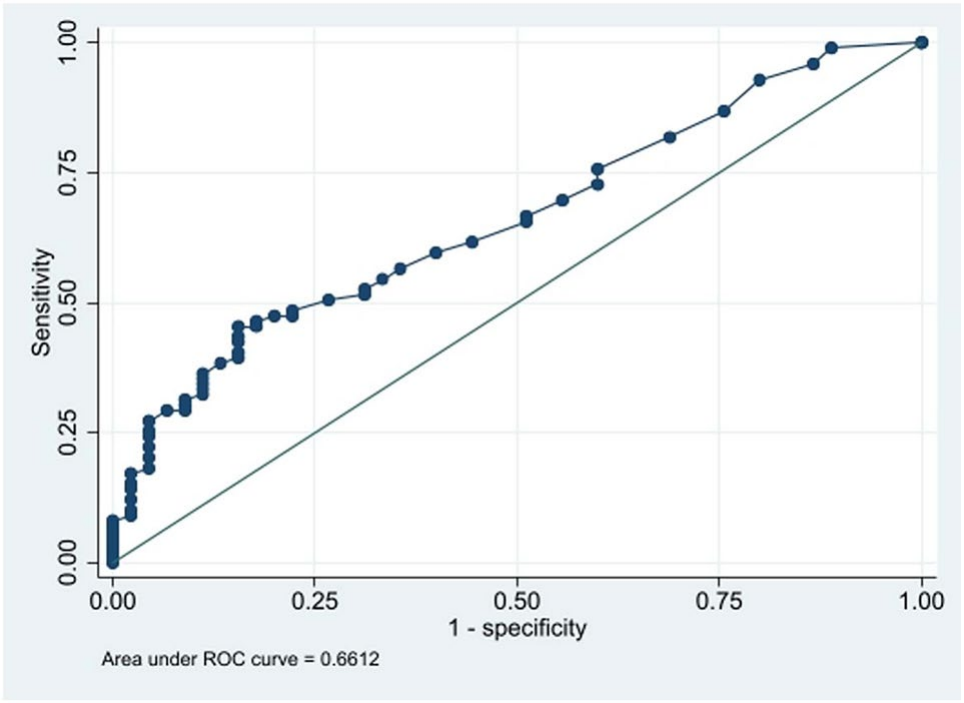
Graph representing the ROC curve of the logistic regression model predicting spontaneous correction of PSI

The logistic regression model had an AUC of 0.66 (95% CI 0.57–0.75) in the ROC analysis. The stepwise analysis of multiple linear regression yielded the equation F(z) = 1/1 + e^–z^, where F(z) was the probability of spontaneous correction of PSI and Z = 2.9854 + 1.0434*postoperative LC + 0.3602*postoperative T1 tilt (Fig. 3).

## Discussion

PSI in AIS patients after corrective surgery can result in cosmetic issues and dissatisfaction, with reoperation being necessary in some cases [1]. Previous investigations found that the incidence of PSI in individuals with scoliosis after corrective surgery varied greatly, ranging from 5.1% to 57.1% [1, 17]. Numerous research papers have examined the associated factors for PSI in AIS patients after corrective surgery. According to Lee et al.’s [8] and Yagi et al.’s [9] study, Risser sign was found to be a risk factor for PSI. Yang et al. [11] found that preoperative PTC, postoperative PTC, postoperative AVT of PTC, and AVT of PTC at follow-up was associated with PSI. Zhang et al. [2] found that postoperative LC was associated with PSI. However, spontaneous correction of PSI has not been previously investigated. Our study aimed to determine its incidence and the associated factors, and in our center, it was not difficult to include only PSI cases since we have quite a large number of PSI cases after corrective surgery for AIS. Our findings revealed that, with a mean follow-up time of 8.47 ± 3.57 months, 68.75% of PSI patients underwent spontaneous correction during the follow-up.

Our study identified postoperative T1 tilt as being associated with spontaneous correction of PSI. The T1 vertebra remains constant relative to spinal alignment, making the T1 tilt a crucial factor in correcting PSI. Conversely, CA and RSH are measured at the shoulder region, which involves the scapulothoracic joint, indicating that scapulothoracic muscle training can potentially normalize CA and RSH no matter whether they are high or low. Clinically, postoperative AIS patients with PSI can train their shoulders by visualizing their shoulder in the mirror and then trying to adjust the imbalanced shoulder.

Our study also found that increased postoperative LC was associated with the spontaneous correction of PSI. This finding may be attributed to the lumbar segment's mobility, which could compensate for and correct shoulder imbalance during the follow-up period. Conversely, PSI resulting from other curves, such as PTC, tends to be stiffer, making spontaneous correction of the imbalance less likely.

This study has several limitations. First, the maximum follow-up time was only 1 year, potentially limiting the detection of spontaneous correction of shoulder imbalance beyond this period. Second, the study was solely radiographic and did not consider the clinical aspect of patients' shoulder balance. It is not necessary that a patient with a radiologic imbalance always have a clinical imbalance. Finally, as the lumbar mobility was preserved in only Lenke types 1, 2, and some of types 3 and 4 that underwent surgery, further investigations may be required to determine the effect of LC on each Lenke type associated with spontaneous correction of PSI.

## Conclusions

The incidence of spontaneous correction of PSI was high (68.75%) during the follow-up period. Postoperative T1 tilt was significantly associated with spontaneous correction of PSI, as patients with normal postoperative T1 tilt values had a higher likelihood of spontaneously correcting shoulder imbalance. Additionally, postoperative LC was significantly associated with the spontaneous correction of PSI, as patients with more postoperative LC were associated with the spontaneous correction of PSI.

## Data Availability

The datasets used and/or analysed the current study are available from the corresponding author on reasonable request.

## References

[1] Smyrnis, P. N., Sekouris, N., & Papadopoulos, G. (2009). Surgical assessment of the proximal thoracic curve in adolescent idiopathic scoliosis. *European Spine Journal,**18*, 522–530. 10.1007/s00586-009-0902-3 19219467 10.1007/s00586-009-0902-3 PMC2899467

[2] Zhang, S., Zhang, L., Feng, X., & Yang, H. (2018). Incidence and risk factors for postoperative shoulder imbalance in scoliosis: A systematic review and meta-analysis. *European Spine Journal,**27*, 358–369. 10.1007/s00586-017-5289-y 28889339 10.1007/s00586-017-5289-y

[3] Bagó, J., Carrera, L., March, B., & Villanueva, C. (1996). Four radiological measures to estimate shoulder balance in scoliosis. *Journal of Pediatric Orthopedics. Part B,**5*, 31–34. 10.1097/01202412-199605010-00006 8744429 10.1097/01202412-199605010-00006

[4] Yang, S., Feuchtbaum, E., Werner, B. C., Cho, W., Reddi, V., & Arlet, V. (2012). Does anterior shoulder balance in adolescent idiopathic scoliosis correlate with posterior shoulder balance clinically and radiographically? *European Spine Journal,**21*, 1978–1983. 10.1007/s00586-012-2434-5 22842954 10.1007/s00586-012-2434-5 PMC3463704

[5] Kuklo, T. R., Lenke, L. G., Graham, E. J., Won, D. S., Sweet, F. A., Blanke, K. M., et al. (2002). Correlation of radiographic, clinical, and patient assessment of shoulder balance following fusion versus nonfusion of the proximal thoracic curve in adolescent idiopathic scoliosis. *Spine (Phila Pa 1976),**27*, 2013–2020. 10.1097/00007632-200209150-00009 12634561 10.1097/00007632-200209150-00009

[6] Wei Chan, C. Y., Chiu, C. K., Ng, Y. H., Goh, S. H., Ler, X. Y., Ng, S. J., et al. (2020). An analysis of preoperative shoulder and neck balance and surgical outcome in 111 adolescent idiopathic scoliosis patients with two subtypes of Lenke 1 curves. *Journal of Neurosurgery. Spine,**34*, 37–44. 10.3171/2020.5.Spine20397 32858516 10.3171/2020.5.SPINE20397

[7] Akel, I., Pekmezci, M., Hayran, M., Genc, Y., Kocak, O., Derman, O., et al. (2008). Evaluation of shoulder balance in the normal adolescent population and its correlation with radiological parameters. *European Spine Journal,**17*, 348–354. 10.1007/s00586-007-0546-0 18027001 10.1007/s00586-007-0546-0 PMC2270384

[8] Lee, C. S., Hwang, C. J., Lim, E. J., Lee, D. H., & Cho, J. H. (2016). A retrospective study to reveal factors associated with postoperative shoulder imbalance in patients with adolescent idiopathic scoliosis with double thoracic curve. *Journal of Neurosurgery. Pediatrics,**25*, 744–752. 10.3171/2016.6.Peds16162 27662445 10.3171/2016.6.PEDS16162

[9] Yagi, M., Takemitsu, M., & Machida, M. (2013). Chest cage angle difference and rotation of main thoracic curve are independent risk factors of postoperative shoulder imbalance in surgically treated patients with adolescent idiopathic scoliosis. *Spine (Phila Pa 1976),**38*, E1209-1215. 10.1097/BRS.0b013e31829e0309 23759803 10.1097/BRS.0b013e31829e0309

[10] Cao, K., Watanabe, K., Hosogane, N., Toyama, Y., Yonezawa, I., Machida, M., et al. (2014). Association of postoperative shoulder balance with adding-on in Lenke Type II adolescent idiopathic scoliosis. *Spine (Phila Pa 1976),**39*, E705-712. 10.1097/brs.0000000000000325 24718061 10.1097/BRS.0000000000000325

[11] Yang, Y., Yang, M., Zhao, J., Zhao, Y., Yang, C., & Li, M. (2019). Postoperative shoulder imbalance in adolescent idiopathic scoliosis: Risk factors and predictive index. *European Spine Journal,**28*, 1331–1341. 10.1007/s00586-019-05933-2 30949769 10.1007/s00586-019-05933-2

[12] O’Brien MF, Kuklo TR, Blanke KM LL. Spinal Deformity Study Group’s Radiographic Measurement Manual.: Memphis: Medtronic Sofamor Danes Inc; 2008.

[13] Kuklo, T. R., Potter, B. K., Schroeder, T. M., & O’Brien, M. F. (2006). Comparison of manual and digital measurements in adolescent idiopathic scoliosis. *Spine (Phila Pa 1976),**31*, 1240–1246. 10.1097/01.brs.0000217774.13433.a7 16688038 10.1097/01.brs.0000217774.13433.a7

[14] Kwan, M. K., & Chan, C. Y. (2016). Is there an optimal upper instrumented vertebra (UIV) tilt angle to prevent post-operative shoulder imbalance and neck tilt in Lenke 1 and 2 adolescent idiopathic scoliosis (AIS) patients? *European Spine Journal,**25*, 3065–3074. 10.1007/s00586-016-4529-x 27021616 10.1007/s00586-016-4529-x

[15] Li, J., Hwang, S. W., Shi, Z., Yan, N., Yang, C., Wang, C., et al. (2011). Analysis of radiographic parameters relevant to the lowest instrumented vertebrae and postoperative coronal balance in Lenke 5C patients. *Spine (Phila Pa 1976),**36*, 1673–1678. 10.1097/BRS.0b013e3182091fba 21358470 10.1097/BRS.0b013e3182091fba

[16] Bravo, G., & Potvin, L. (1991). Estimating the reliability of continuous measures with Cronbach’s alpha or the intraclass correlation coefficient: Toward the integration of two traditions. *Journal of Clinical Epidemiology,**44*, 381–390. 10.1016/0895-4356(91)90076-l 2010781 10.1016/0895-4356(91)90076-l

[17] Menon, K. V., Pillay, H. M., & Tahasildar, M. A. N. (2015). Post-operative shoulder imbalance in adolescent idiopathic scoliosis: a study of clinical photographs. *Scoliosis,**10*, 31. 10.1186/s13013-015-0055-6 26582232 10.1186/s13013-015-0055-6 PMC4650280

